# Efficacy Comparison Between Interscalene Block with and Without Superficial Cervical Plexus Block for Anesthesia in Clavicle Surgery

**DOI:** 10.5812/aapm-142051

**Published:** 2024-01-20

**Authors:** Faramarz Mosaffa, Mahshid Ghasemi, Afsaneh Habibi, Reza Minaei, Narges Bazgir, Elham Memary, Alireza Shakeri

**Affiliations:** 1 Anesthesiology Research Center, Shahid Beheshti University of Medical Sciences, Tehran, Iran; 2 Akhtar Orthopedic Research Center, Shahid Beheshti Medical University, Tehran, Iran; 3 Hearing Disorders Research Center, Loghman Hakim Hospital, Shahid Beheshti University of Medical Sciences, Tehran, Iran

**Keywords:** Clavicle Surgery, Interscalene Block, Superficial Cervical Plexus Block

## Abstract

**Background:**

Clavicle fractures account for over one-third of shoulder injuries and up to 3.3% of all fractures in adults. While the majority of these fractures can be managed non-surgically, there are instances where surgical intervention is performed. Regional anesthesia (RA) can be a preferred alternative to general anesthesia (GA) to avoid complications and high costs in this surgery. Moreover, the identification of the most optimal approach for RA remains challenging.

**Objectives:**

This study aimed to compare the efficacy of interscalene block (ISB) with and without superficial cervical plexus block (SCPB) as an anesthetic technique for clavicular fracture operation.

**Methods:**

This double-blinded, non-inferiority clinical trial was conducted on 120 patients randomly divided into 2 groups: One receiving ISB and the other receiving ISB with SCPB. The primary outcome was defined as the conversion to GA. Various factors were recorded, including surgery duration, nerve block initiation, analgesics required in the postanesthesia care unit (PACU), and sedation during surgery. Pain was evaluated using the Visual Analog Scale (VAS) in PACU. SPSS version 26 was used for statistical analysis, performing descriptive analysis, Student’s *t*-tests, and Mann-Whitney U tests to compare non-parametric variables between the 2 groups. Statistically significant results had a P value of less than 0.05.

**Results:**

A total of 120 patients were randomly divided into 2 equal groups, each consisting of 50 males and 10 females. The mean age of intervention and case groups were 37.23 ± 13.30 and 38.43 ± 11.95 years, respectively. After performing statistical tests (Student's *t*-test and Mann-Whitney U test), there was no significant difference in the initiation time of nerve block, surgery initiation time, surgery duration, the amount of required sedation, VAS scores, and meperidine consumption (P > 0.05). None of the patients in both groups required conversion to GA.

**Conclusions:**

The primary goal was achieved in all included cases, and no patients required conversion to GA. The efficacy of ISB is the same whether or not it is combined with a SCPB. Interscalene block is an alternative RA approach for clavicle fractures. Thus, ISB alone is as efficient as when used in combination with SCPB.

## 1. Background

Clavicle fractures make up 44% of all shoulder girdle fractures and 2.6% - 3.3% of all orthopedic fractures (1, 2). Clavicle fractures usually occur after blunt traumas. The majority of clavicle fractures are successfully managed conservatively (3). The surgical indication for a clavicle fracture is more than 2 cm displacement (4-6). In shortening more than the mentioned amount, open reduction and internal fixation (ORIF) are used (4, 7, 8).

**Figure 1.**
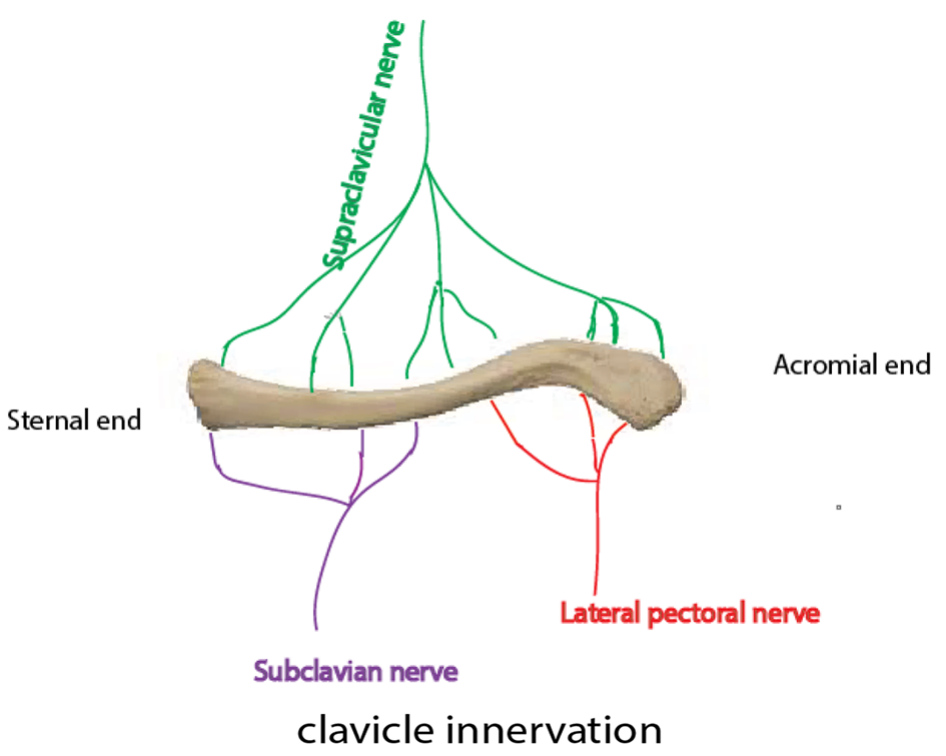
The innervation of the clavicle

Due to the complex innervation of the clavicle by subscapular, supraclavicular, and subclavian nerves (Figure 1), ORIF is commonly performed under general anesthesia (GA) (8).

The complex innervation of the clavicle and interpersonal differences between patients in clavicular innervation are the major concerns for regional anesthesia (RA) (8).

Although GA provides complete immobility, increased airway adverse events, elevated risk of hemodynamic stresses, nausea, and vomiting are among its side effects (9, 10). On the other hand, RA can prevent unwanted side effects of GA and provide better analgesia and faster recovery and movement for the patient (9). The lower cost of RA is also one of its other benefits (11, 12).

Understanding the innervation of the clavicle is crucial for designing effective RA techniques. The supraclavicular nerve provides sensory innervation to the clavicular skin, while the clavicle itself is innervated by several nerves (Figure 1). The ventral and cephalic parts are supplied by supraclavicular nerves. The innervations of the caudal and dorsal parts of the clavicle are provided by both the subclavian and lateral pectoral nerves. The sensory innervations of the clavicle medial and lateral parts are provided by spinal accessory and subscapular and axillary nerves, respectively (8, 13, 14).

Many studies have suggested interventional strategies of RA for clavicular fractures, including superficial cervical plexus block (SCPB), interscalene block (ISB), and combined superficial cervical-deep cervical plexus (8). Regarding the neuroanatomy and clinical experiences, the combined interscalene-cervical block in clavicular fracture surgeries provides sufficient analgesia. Thus, regarding clavicle innervation, we hypothesized that ISB may be as efficient as the combination of ISB and SCPB.

## 2. Objectives

This study aimed to compare the efficacy of ISB with and without SCPB as an anesthesiologic technique for clavicular fracture operation.

## 3. Methods

A randomized, double-blinded, non-inferiority clinical trial was conducted in Akhtar Hospital from March to June 2023. Patients with clavicle fractures aged 18 to 60, weighing less than 80 kg and consenting to participate in the study were included. Included patients must have had normal neuromotor and sensory function. Patients treated with narcotics or had a history of alcohol, narcotics, or any addictive drug abuse were excluded. Other exclusion criteria included any contraindication of nerve blocks like local infections, coagulopathy, and any allergy to local anesthetics.

Furthermore, patients with mental disorders, restrictive pulmonary disease, pregnancy, bradycardia (heart rate less than 50), and those who consumed beta blockers were all excluded. A complete history was obtained from each case. All the included individuals underwent a thorough neurological examination.

The Ethics Committee of Shahid Beheshti University of Medical Sciences approved this survey (code: IR.SBMU.MSP.REC.1401.526, date of registration: 25/01/2023; the Iranian Registry of Clinical Trials. code: IRCT20230204057318N1). The study protocol followed the Declaration of Helsinki. All the included patients were informed about the purpose of the study, and written consent was obtained.

G power software was used to determine the sample size. Based on Ryan et al.’s study (15), by considering the effect size = 0.54, α = 0.05, and β (Power) = 0.90, 120 patients for the sample size were chosen for the sample size. Afterward, patients were randomly divided into 2 groups. To ensure fairness, we used computer-generated software to create equal groups through a randomized method. An assistant who was not associated with this study was responsible for generating and managing a random sequence. The randomization code was placed in a sealed opaque envelope by the assistant. A total of 120 cards were selected, with 60 cards in each group. The cards were placed in sealed opaque envelopes and shuffled. Patients selected envelopes without knowledge of the contents. Only those responsible for the randomization process knew which cards were in each envelope, while other members of the research team were kept unaware. After enrolling a patient in the study, an anesthesiologist opened the sealed envelope and proceeded with the allocated procedure. To prevent accidental revelation of allocation to the participants, the block needle is introduced through the same area for both interventions. Additionally, the ultrasound monitor was not within the participant's line of sight. After the procedures, the researcher who assessed the outcome was unaware of the randomization. In case of serious adverse events that could potentially harm the participants, the patients were immediately removed from the trial. The blinding was also removed, and the events were reported. Additionally, the data analyst was also kept unaware of the randomization process.

**Figure 2.**
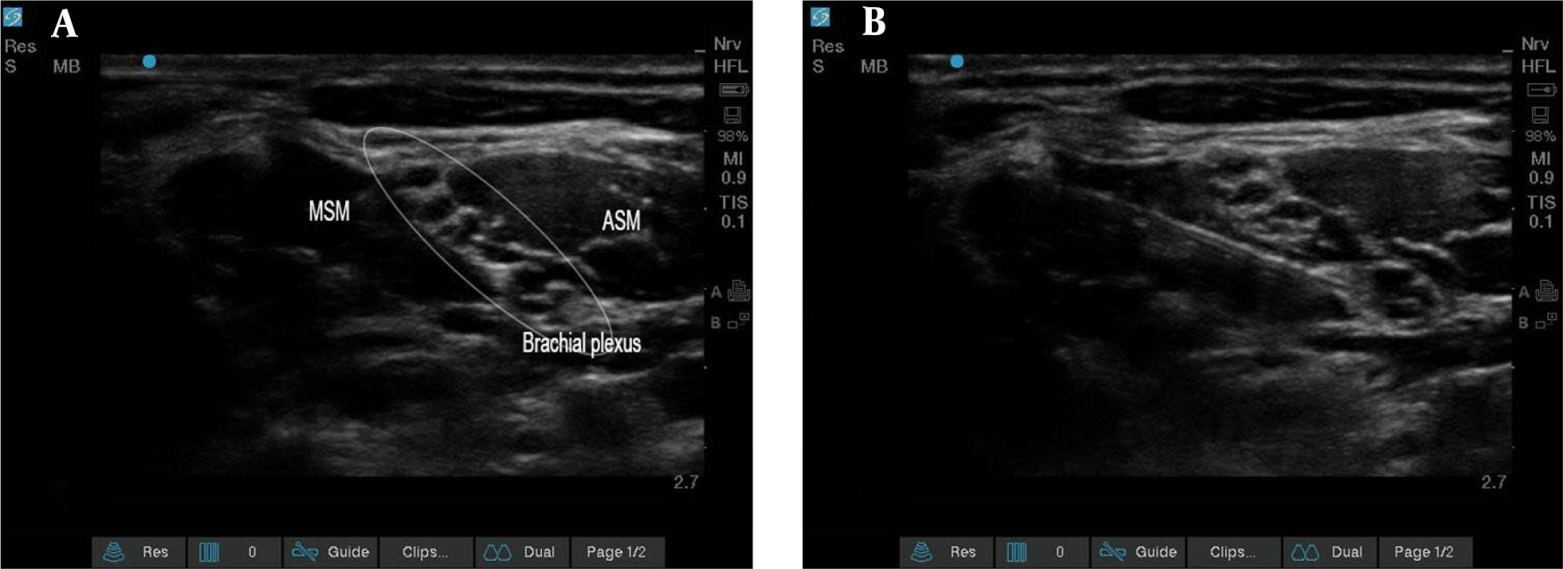
Sonography of the brachial plexus, middle scalene muscle, and anterior scalene muscle. The b and a are before and after the injection, respectively (Abbreviations: MSM, middle scalene muscle; ASM, anterior scalene muscle).

In the block room, all patients underwent standard monitoring (pulse oximetry, noninvasive blood pressure, and electrocardiogram). In the first group, after placing patients in a supine position with a 30° head-up and dis-infecting the neck with povidone-iodine, ISB was performed by a single anesthesiologist who is an expert in RA (Figure 2). 

Initially, the brachial plexus was identified between the anterior and middle scalene muscles. Then, via ultrasound visualization, a needle was inserted from lateral to medial into the interscalene grove between the nerve roots. Consequently, 20 mL of 1.5% lidocaine (Caspin Company), 1 mL of 8.4% bicarbonate (Caspin Company), 1: 200 000 epinephrine (Daroupakhsh Company), and 4 mL of 0.5% bupivacaine (AstraZeneca Company) were used.

**Figure 3.**
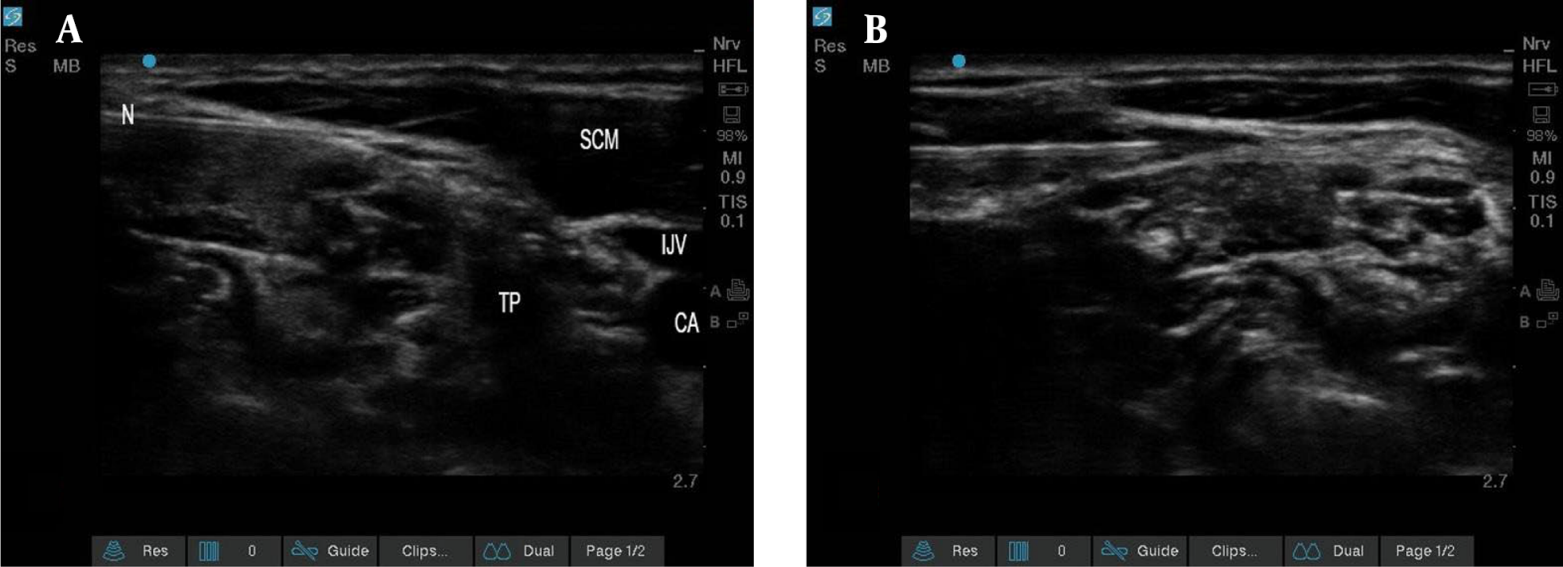
The sternocleidomastoid, internal jugular vein, carotid artery, transverse process, and needle. The A and B are before and after the injection, respectively (Abbreviations: SCM, sternocleidomastoid; IJV, internal jugular vein; CA, carotid artery; TP, transverse process; N, needle).

In addition to the mentioned ISB method, SCPB was performed. The high-frequency linear ultrasound probe was slid to the lateral side of the neck at the midpoint of the sternocleidomastoid (SCM) muscle, corresponding to the C6 transverse apophysis and its anterior tubercle at the level of the cricoid cartilage. After identifying the muscle, the probe was moved backward until the posterior edge of the muscle was found. The interscalene groove between the anterior and middle scalene muscles was then identified. Next, SCP was located just above the prevertebral fascia that covers the interscalene groove enclosed within the interstitial space, separating the cervical fascia and the posterior sheath of SCM. A block needle was then introduced from lateral to medial using the posterior-in-plane technique till its tip was placed near the SCP above the prevertebral fascia. After careful negative aspiration to exclude intravascular placement, 10 mL of 1.5% lidocaine (Caspin Company), 1 mL of 8.4% bicarbonate (Caspin Company), 1: 200 000 epinephrine (Daroupakhsh Company), and 4 mL of 0.5% bupivacaine (AstraZeneca Company) were deposited (Figure 3). 

In both groups, nerve blocks were performed by a single anesthesiologist who is an expert in RA under an ultrasonographic guide (S-Nerve ultrasound Sonosite machine) with a linear transducer with 6 - 15 MHz frequency using the in-plane method with a block needle (B. Braun needle, 22Ga, 80 mm, Stimuplex Ultra 360).

All common challenges of nerve blocks, such as nerve injury, vascular injury, local anesthesia toxicity, and the absence of neurological lesions and neurological evaluation after nerve blocks, were considered for safety considerations.

Loss of shoulder abduction was used to identify motor blockade, while the pinprick test and palpitation were used to determine sensory blockade at the operation site. Also, the arm mobilized passively to assess further pain. A block was deemed successful if all the examinations, as mentioned earlier, were present. If uncontrolled pain occurred after starting surgery that required conversion to GA, the block was considered incompetence.

In the case of patient anxiety, 2 mg of midazolam was administered, and if a patient complained of pain, 50 μg of fentanyl was administered intravenously. If adverse reactions, like bradypnea (respiratory rate less than 8), apnea of more than 15 s, and oxygen saturation of less than 94%, were observed, the process was held, and patients' respiration was aided. At the end of the operation, patients were transferred to the postanesthesia care unit (PACU).

Our primary goal was to compare the need for inducing GA for the operation between intervention and control groups. The secondary outcomes included the requirement for sedation during surgery, comparing postoperative Visual Analog Scale (VAS) scores, and the amount of narcotics used in the PACU between the 2 groups.

Demographic data, the duration of surgery, the beginning of the nerve block, the surgery initiation time, the amount of required sedation during surgery, and the amount of required analgesics in the PACU (meperidine) were all thoroughly recorded. The meperidine was used in fixed doses of 20 mg, repeated every 5 min to achieve adequate response. We also evaluated the pain experienced by patients after the VAS operation in the PACU. All included individuals were followed up until their discharge from the PACU.

The group-matching method was used to omit the confounders. Selection, performance, and detection biases were all omitted by randomization and blinding, respectively. We reported all the significant and insignificant findings of the study. The results were reported completely and clearly. Initially, 7 patients did not consent to receive RA, but no patient was lost during follow-up. To minimize the random error, the sample size was carefully determined prior to the study.

To remove the biases from the randomization process, the allocation was random, and the allocation sequences were concealed. Patients were unaware of the allocation. The main researchers and the expert regional anesthesiologist were the only people who were aware of randomization. The data of all included cases were available, and we had no missing data. Furthermore, the method of measurement was the same in both groups, and the outcome assessor was not aware of allocation. Afterward, data were completely gathered, and all of them were transferred to a blinded data analyst.

The statistical analysis was conducted using SPSS version 26. Descriptive analysis and Students' *t*-tests were performed. The Mann-Whitney U test was conducted to compare non-parametric variables between the 2 groups. P values less than 0.05 were considered statistically significant.

## 4. Results

We conducted a double-blinded clinical trial in Akhtar Hospital. Initially, 136 patients were assessed for eligibility. Seven patients did not consent to enroll in the study. A hundred and twenty-nine patients with clavicle fractures were included in this survey (64 patients in the ISB + SCPB group and 65 in the ISB group). Nine patients were excluded due to an unsuccessful shoulder motor block. Thus, 120 patients remained (60 in each group). Further, during the survey, no individual was excluded. Thus, all primary and secondary outcomes were evaluated for 60 participants in each group.

Each group comprised 10 females (16.75%) and 50 males (83.3%). The mean age of intervention and case groups were 37.23 ± 13.30 and 38.43 ± 11.95 years, respectively. After conducting the Students' *t*-test, there was no significant difference in mean ages between the 2 groups (P = 0.604).

No case in either group required conversion to GA. The mean and SD of surgery duration, nerve block initiation time, and surgery initiation time are listed in Table 1. 

**Table 1.** Means, SDs, and the Resulting P Values from Student’s *t*-Test ^a^

Variables	Combined Interscalene and Cervical Plexus Block Group (n = 60)	Interscalene Group (n = 60)	P-Value
**Duration of surgery (min)**	66.57 ± 7.37	65.87 ± 6.29	0.557
**Nerve block initiation (min)**	10.63 ± 1.96	11.15 ± 2.07	0.276
**Surgery initiation time (min)**	22.33 ± 2.81	22.02 ± 2.81	0.474

^a^ Values are presented as mean ± SD.

A Student's *t*-test was conducted to compare these variables. As shown in Table 1, the nerve block initiation was slightly lower in patients who received ISB and SCPB (10.63 ± 1.96 vs 11.15 ± 2.07 min). However, this difference was not statistically significant. Besides, no significant differences were observed between the 2 groups in surgery duration, the time of nerve block initiation, and surgery initiation time (P > 0.05). The means and SDs of these variables and resultant P values are demonstrated in Table 1. 

As demonstrated in Table 2, there was no significant difference between sedation requirements and the amount of meperidine consumption in PACU between the 2 groups.

**Table 2.** Sedation Needs and Analgesic in the Postanesthesia Care Unit and the Resulting P-Values from the Mann-Whitney U Test ^a^

Variables	Combined Interscalene with Cervical Plexus Block Group (n = 60)	Interscalene Group (n = 60)	P-Value
**Fentanyl 50 mcg**	5 (8.3)	6 (10.0)	0.752
**Midazolam 2 mg**	11 (18.3)	10 (16.7)	0.810
**Analgesic in PACU**	4 (6.7)	4 (6.7)	> 0.999
**Meperidine 20 mg**	4 (6.7)	4 (6.7)	> 0.999

Abbreviation: PACU, postanesthesia care unit.

^a^ Values are presented as No. (%).

**Figure 4.**
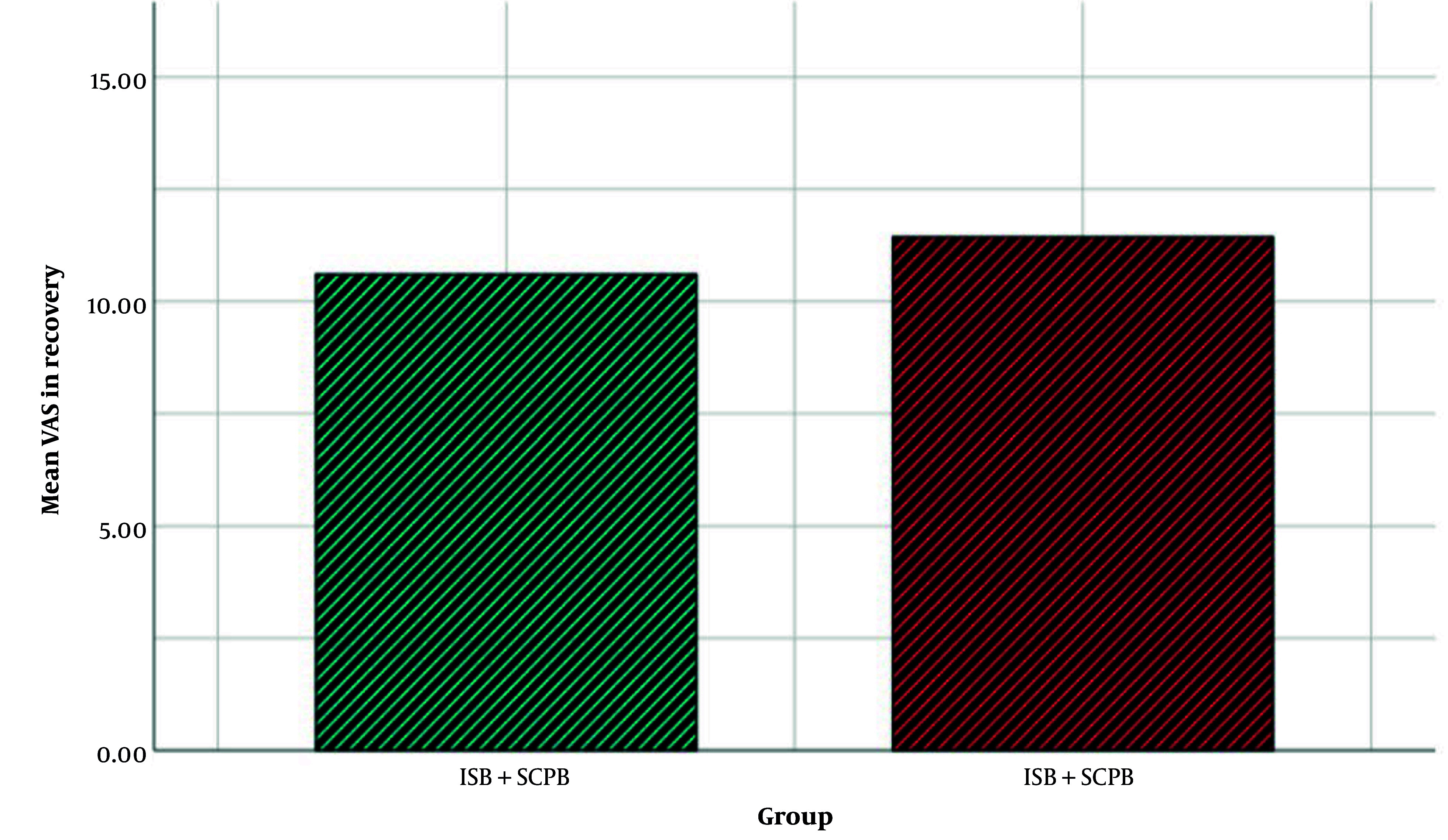
The bar chart of the Visual Analog Scale in the 2 groups. The interscalene block (ISB) + superficial cervical plexus block (SCPB) group received combined interscalene with CPB. The ISB group received ISB (Abbreviations: VAS, Visual Analog Scale; ISB, interscalene block; SCPB, superficial cervical plexus block).

Furthermore, the VAS means of ISB and ISB + SCPB were 11.43 ± 4.25 and 10.6 ± 4.28, respectively. After analyzing the VAS means with the Mann-Whitney U test, it was revealed that there was no significant difference between the 2 groups (P = 0.338). Figure 4 shows the mean of VAS in the 2 groups.

## 5. Discussion

This article aimed to compare the efficacy of ISB with and without SCPB as an alternative RA approach for clavicle fractures. As demonstrated, there was no significant difference between the 2 groups in surgery duration, onset of nerve block, onset of surgery, VAS scores, amount of required sedation, and meperidine consumption in the PACU. No complications were recorded in the 2 groups. No case required conversion to GA and all the operations were performed successfully.

Several previous studies have proved that RA and GA had the same efficacy. In addition, Ryan et al. retrospectively reviewed 110 clavicle fractures and demonstrated that the average operation time of patients who underwent RA was 30 minutes shorter than patients with GA. Since the estimated cost of surgery in their center was $60/min, RA decreased the time of operation and the costs significantly (15).

A study compared 2 groups of patients with clavicle fractures. One group received GA, while the other received a combination of nerve blocks. The second group required less opioid medication and had a shorter stay in the PACU (16). Similarly, patients with clavicle fractures who received RA had less pain and consumed fewer opioids than those who received GA. Cervical and interscalene brachial plexus blocks resulted in minimal pain and opioid use (17).

Consequently, RA approaches were as efficient as GA. Also, RA methods have fewer side effects and a lower need for sedation postoperatively.

Fugelli et al. evaluated 10 patients with midshaft clavicle fractures who received ISB + SCPB. They showed that all patients successfully underwent clavicle surgery with no need for GA conversion. They observed no adverse events in their cases. Accordingly, our cases had no side effects, and none of our cases required conversion to GA (18).

A clinical trial compared ultrasound-guided ISB with SCPB and ISB with intermediate cervical plexus block (ICPB) for clavicle surgery in 50 patients divided into 2 groups. Group 1 had higher nerve block success with ISB and ICPB, while group 2 experienced delayed onset and shorter nerve block duration; ISB with ICPB was a better anesthetic approach for clavicle surgery (13). The sensory block initiation in this study for patients who received ISB and SCPB was 4.3 ± 0.5 min, which was clearly less than the nerve block initiation in our survey (10.63 ± 1.96 min). The difference is that Arjun et al. evaluated the sensory block while we measured the motor nerve block initiation. Furthermore, the type and amount of analgesics used in our survey differed from those in Arjun et al. (13).

A randomized clinical trial was conducted by Gupta et al. with 60 patients divided into 2 groups. One group received cervical plexus blockage and ISB, while the other only received ISB. A nerve locator was used to perform the nerve block. The latter group required extra RA for over a quarter of patients, and about 10% needed conversion to GA (14). In contrast, none of our cases needed more local anesthesia or conversion to GA. Although the supraclavicular nerve was not directly anesthetized, all individuals in our study reached sufficient anesthesia for clavicle surgery. This difference may be due to diffusion of the higher volume of local anesthetic we used, thus affecting the supraclavicular nerve, making ISB sufficient. Our study had a larger sample size, leading to more precise results, making our study superior to Gupta et al. Besides, we also used sonography instead of a nerve locator for nerve block (14).

Abdelghany et al. included 70 patients with clavicle fractures who were candidates for internal fixation. These patients were equally divided into 2 groups. One receiving SCPB and the other ISB + SCPB. It was revealed that the incidence of phrenic nerve palsy was less in the group that received SCPB. As a result, they concluded that SCPB is as efficient as ISB and SCPB and it has fewer complications (19). Likewise, the fentanyl consumption between the 2 groups in Abdelghany et al.’s study was the same. Compared to this study, the duration of surgery in ours was shorter. However, the VAS score in the current study was much higher. The reason for the higher VAS score is that the VAS was measured immediately in the PACU, while in Abdelghany et al.’s study, the VAS was measured after 2 h. Furthermore, the type of the anesthetic and the dosage varied between the 2 studies (19).

New methods have been developed to provide more targeted pain relief. A clinical trial conducted by Han et al. looked into the effectiveness of 2 different nerve block methods on 91 patients with medial shaft and medial clavicle fractures. The participants were divided into 2 groups: one group received C3, C4, and C5 nerve blocks using 5 mL of 0.5% ropivacaine; in contrast, the other group received ISB combined with ICPB using 20 mL of 0.5% ropivacaine under ultrasound guidance. The study found that C3, C4, and C5 nerve blocks were more effective than ISB combined with ICPB. Patients who underwent C3, C4, and C5 nerve blocks experienced a quicker onset of sensory block, and the duration of the sensory block was longer than in the other group. Many patients in the C3, C4, and C5 groups also had successful nerve blocks. However, the 2 groups had no difference in the Numeric Rating Scale (NRS). Therefore, C3, C4, and C5 nerve blocks are a suitable RA method for middle clavicle fractures (20). Consequently, this study shows that RA approaches, which are more selective and less extensive, are as efficient as the conventional methods.

In a case series with 20 patients with clavicle fractures, the C5 root and supraclavicular nerve block were used. However, 4 patients experienced inadequate anesthesia, 3 needed further sedations, and 1 had to undergo GA. This could be attributed to the complex nerve supply of the clavicle. To prevent such occurrences, exploring alternative methods that require reduced anesthesia and offer superior outcomes compared to GA would be advantageous (21). Less extensive RA approaches are preferable due to fewer complications and lower costs. However, proper nerve paralysis is necessary to avoid conversion to GA and resulting complications.

### 5.1. Limitations

This study has several limitations. First, we did not consider the classifications of clavicle fractures. Second, due to the high incidence of clavicle fracture in the younger population, most included patients were young with good medical status. The analgesic effects were evaluated until the discharge from the PACU. A longer follow-up duration for recording analgesic effects and side effects is required. A larger multi-centric clinical trial with a longer follow-up duration should be performed to obtain results that are more precise.

### 5.2. Conclusions

No significant differences were found in surgery duration, nerve block initiation time, surgery initiation time, sedation requirements, or meperidine consumption in the PACU between the 2 groups. The VAS means of the ISB and ISB + SCPB groups were not significantly different between the 2 groups. The rate of the conversion to GA did not differ between the 2 groups. As a result, it was found that ISB was as efficient as SCPB in combination with clavicle fracture surgery.

## Data Availability

All analyzed data during this study are included in this article. Further inquiries can be directed to the corresponding author.

## Clinical Trial Registration Code:

IRCT20230204057318N1.
